# An attachment-based framework for disordered personality development: Implications for intersubjective psychodynamic psychotherapy

**DOI:** 10.3389/fpsyt.2022.970116

**Published:** 2022-10-18

**Authors:** Paolo Brambilla, Cinzia Bressi, Bruno Biagianti

**Affiliations:** ^1^Department of Neurosciences and Mental Health, Fondazione IRCCS Ca' Granda Ospedale Maggiore Policlinico, Milan, Italy; ^2^Department of Pathophysiology and Transplantation, University of Milan, Milan, Italy

**Keywords:** attachment, intersubjectivity, psychodynamic psychotherapy, personality development, personality disorders

## Abstract

Infant-caregiver dyads show high heterogeneity in terms of compatibility. Several lines of evidence indicate that the modalities by which areas of good and poor fit were emotionally recognized and managed by caregivers influence the infant's personality development, the integration of their personality traits, the overall sense of authenticity, as well as the modalities of transference that typically manifest during psychodynamic psychotherapy. Within an intersubjective framework, the relationship between patient and psychotherapist will inevitably recreate compatibility issues, although the specific areas of incompatibility will likely differ from the scenarios present in the caregiver relationship. In other words, emotional friction may originate from personality traits that were not problematic in the first place. The author hypothesizes that disclosure of the challenges associated with the management of areas of incompatibility will not only promote emotional honesty within the dyad, but also offer an excellent opportunity for introjection. Such disclosures are not at risk of being interpreted as an attempt to build an intersubjective experience, but represent a window into authenticity, which in turn enables patients to develop awareness of their personality and relational traits, along with the challenges and vulnerabilities that occur when such traits interface with otherness.

## Compatibility in the infant-caregiver dyad and personality development

Several lines of evidence for psychological and biological research indicate that every person is born with biopsychological traits (1, 2). These traits are unique modalities that strongly contribute to how the individual perceives, processes, and expresses emotions; responds to interpersonal stimuli, behaves socially, and manages inner and outer conflict; and reflects, develops, and ultimately communicates thoughts (3).

As newborns are entirely dependent on their caregivers, the early relational environment greatly influences the emergence, paths of development, and possible impairment of such traits (4). Whereas the role and/or the intention of a caregiver is to make room for the development of their infant's authentic traits, to perceive their wishes and needs, and to adapt to them, it is undeniable that every caregiver is primarily an individual who carries their own biopsychological traits. Therefore, there is a *pre-*intentional, non-verbal level where infant and caregiver interact on an equal footing, and continuously perceive areas of compatibility or incompatibility as the relationship evolves (5). Notably, although a compatible trait likely represents an opportunity for harmony, it could also generate friction if the infant has a negative identification with the trait. As a result, depending on the intrinsic traits that the infant and caregiver carry, a lesser or greater degree of compatibility may occur (6). For example, an infant may experience emotions in an energetic, intense, expansive, rapid-onset, and rapid-metabolism manner. If the caregiver perceives emotions in a similar way, an instinctual understanding will likely open between them, one based on emotional resonance, i.e., identification (7–10). Conversely, if the caregiver has, for example, a soft, slow, and private way of processing emotions, this could easily generate in both caregiver and infant a non-verbal sense of emotional otherness, which could produce friction (11).

Although it can be assumed that the caregiver has experienced otherness in many ways throughout their life, and has developed their own response to it, the experience of pregnancy and possibly nursing can pose exceedingly hard challenges for mothers, who must navigate the complex transition from oneness to otherness–a separation that is no less psychological than physical (12). As much as responding to emotional otherness is a learnt behavior accumulated through life experiences (13–15), because individuals are increasingly exposed to complex interpersonal scenarios, that response is no less influenced by biopsychological traits (16). Although some individuals are aversive to emotional otherness, specific kinds of otherness can be naturally appealing for others (17). When emotional otherness is experienced between caregiver and infant, it could elicit different responses (18, 19), from attraction (“I like how you emote”) to emotional friction (“I do not resonate with how my baby feels”; “My caregiver does not resonate with how I feel”).

Areas of compatibility within the dyad—whether originating from resonance or attraction—likely generate harmony between the infant and the caregiver. The caregiver tends to respond favorably to them, letting them permeate the relationship and become positive identification opportunities for the infant (20). Areas of incompatibility, on the other hand, can generate emotional friction—to which caregiver and infant can respond very differently (21). Although a caregiver has putatively developed cognitive resources they can mobilize to handle friction, an infant likely has not reached the necessary milestones of neurodevelopment to do the same (22). Therefore, the emotional management of areas of incompatibility is a responsibility that largely belongs to the caregiver.

If incompatibility evokes feelings of unsuitableness, discomfort, or distress in the caregiver, the infant is likely to perceive it (23). If the caregiver's emotions translate into overt behaviors of fear, avoidance, denial, or judgment, the infant tends to introject the caregiver's reaction, whether or not such reaction is educational or detrimental for their development (24). Common examples can include: considering a part of oneself as “bad” or “dangerous,” repudiating a part of oneself, or denying the presence of emotional and communicative needs because their caregiver is unable to meet them (25). Another option—based on their biopsychological traits, neurodevelopmental stage, and extended relational environment—is for the infant to safeguard the authentic trait that has created the incompatibility, even if that means coping with the absence of harmony with the caregiver (26).

Attachment literature indicates that from a very early developmental stage, infant-caregiver dyads show high heterogeneity in terms of compatibility (27). The validation and integration of the infant's personality traits, the overall sense of authenticity, and the modalities of transference that could manifest during psychodynamic psychotherapy are all heavily influenced by how areas of good and poor fit were emotionally handled in the context of the caregiver relationship (28). Caregivers with greater areas of incompatibility with their infant will therefore need to do more emotional management if they want to promote the normal development of the infant's personality (29). Nonetheless, all individuals carry, to various degrees, the distress that originates from lack of authenticity (not feeling seen for who they truly are), and such distress commonly emerges during psychodynamic psychotherapy (30).

## Compatibility in the patient-therapist dyad during psychodynamic psychotherapy

In a very similar way to that of a caregiver, the role of a “good enough” psychotherapist is to make room for the development of their patients' authentic traits, to perceive their wishes and needs, and to adapt to them (31). However, this encounter exists at multiple levels, including the non-verbal and sensorial one where, from the very first moment and for the entire duration of the therapy, therapist and patient interface as two symmetrical individuals, and mutually experience emotional harmony and friction that originate from areas of good and poor fit (32, 33).

Though psychodynamic psychotherapists were once trained on masking their emotional experiences to facilitate the patient's transference and countertransference (34), there has been increased recognition that, especially during vis-à-vis psychotherapy, their subjectivity is at least partially perceived by patients (35). Whether because of the tone with which they greet or say farewell to their patients, a ritualistic gesture that recurs during sessions, a change in posture or body language, or even the timing of silence, aspects of the person behind the profession—along with all their biopsychological traits—are unequivocally seen (36, 37). In fact, with areas of emotional harmony and friction inevitably emerging from the beginning of psychotherapy, it will come as no surprise that psychotherapists anecdotally speak of patients with whom they have more compatibility as “favorite” patients, and those with more incompatibility as “more difficult” patients (38). Unsurprisingly, in the context of the working relationship developed during short- and long-term psychodynamic therapy, patients find certain characteristics in a therapist helpful, such as basic interpersonal skills, an encouraging relational style, and constructive coping techniques (39, 40). Notably, characteristics that therapists rate as possible predictors of a better patient-rated alliance—such as professional self-confidence, work enjoyment, and self-experiences in personal life—are actually less salient when patients rate that same alliance (41). We posit that the degree to which these viewpoints converge—likely driven by greater intersubjective compatibility—is responsible for patients' and therapists' preference above and beyond social and cultural qualifiers that could differentiate or connect them [e.g., Owen et al. (42)].

## Would recognizing and discussing compatibility benefit the psychotherapy process?

Despite psychotherapists being trained on how to not act upon unpleasant feelings that originate from areas of incompatibility with behaviors that can negatively influence the introjection processes that occur during psychotherapy, should there be a conversation about those feelings? And when should that conversation occur? During the initial phase of psychotherapy, patients are encouraged to freely describe their psychological distress. Through the content that is endorsed session after session, psychotherapists have an excellent opportunity to grasp the biopsychologically driven modalities through which the patient perceives, processes, and expresses emotions, as well as how they reflect, develop, and communicate their thoughts. The psychotherapist will inevitably notice that some of these biopsychological traits are being expressed less authentically (43). Why does that happen? Are these traits that could not develop adequately in the context of the caregiver relationship emerging as inauthenticity in the transference? Has the patient's authenticity been compromised instead because of negative learning experiences unrelated to caregivers, but instead as a result of experiences with peers, cultural surroundings, and associated values? (44). Or has the patient unconsciously detected an area of intersubjective incompatibility, one where unpleasant feelings within the patient-therapist dyad could easily be generated? (45).

In such moments, disambiguation is rather necessary, as is emotional honesty (46). The patient could be asked whether they imagine that the psychotherapist is unlikely to perceive or appreciate the personality trait under scrutiny (47). This allows the dyad to investigate transference and raise awareness of possible projections, while giving the psychotherapist the opportunity to acknowledge that such a trait is perceived, validated, accepted, and, in fact, fully legitimized (48). Above and beyond inevitable transference and countertransference mechanisms, what about the emotional response that the patient's specific trait is eliciting in the person-psychotherapist? Whenever areas of incompatibility are mutually experienced, should psychotherapists be solely preoccupied with successfully managing the emotions associated with that incompatibility (49), or should they disclose their negative response?

Once projections are disentangled from areas of incompatibility, the patient can begin to appreciate the psychotherapist's efforts to overshadow their own personality, and to navigate in a constructive and mature way the areas of friction that are unique to their relationship, as this may not have happened in the context of the caregiver relationship (50). However, the fact that a psychotherapist shows how they successfully manage areas of incompatibility, offering the patient an excellent opportunity for introjection (50) does not preclude that it was emotionally costly to do so, which could be non-verbally signaled and easily detected by the patient (51).

In light of the above, it follows that any relationship between patient and psychotherapist will recreate compatibility issues, except that the specific profile of incompatibility will likely differ from the one that was experienced with caregivers. In other words, emotional friction may originate from personality traits that were not problematic in the first place (52).

In this framework, concepts such as “negative transference,” “unending analysis,” or “still analysis” may stem from the belief that areas of incompatibility within the therapeutic dyad originate from unelaborated experiences on the part of the patient or even the psychotherapist (53), and as such, become therapeutic targets that must be resolved (54, 55). While the patient could aspire to reenacting positive aspects of the therapeutic relationship, they could also feel a need to make changes in response to problematic aspects of the relationship based on areas of incompatibility. Here, the risk is to subject the patient to the process of identify reconfiguration so that they can establish the highest possible degree of adjustment with a figure who, just like the caregiver, is rarely chosen on the basis of compatibility (56, 57).

## Therapeutic alliance as a process of intersubjective negotiation

If and when it is agreed upon that the goal of psychotherapy is not to develop a harmonious relationship with the psychotherapist, but to recognize and further develop the patient's traits, mutual acknowledgment and open discussion of emotional friction within the dyad will become critical milestones in the psychotherapeutic relationship. Scrutinizing the emotional responses will allow to determine whether the perception of incompatibility originates from the patient's intrapsychic conflict or from an actual dyadic intersubjective mismatch. In the first scenario, emotional friction could be interpreted as the result of transference, and therefore psychoanalyzed and worked through (58). In the latter, the emotional cost of intersubjective mismatch should not be subject to interpretation, but instead serve as a window into self-authenticity (59). As a matter of fact, several authors have reconceptualized the therapeutic alliance from a relational perspective as a process of intersubjective negotiation, providing guidelines on how to use self-disclosure and metacommunication as tools to transform ruptures and strains into therapeutic breakthroughs (60). It is precisely thanks to the relational experience with the psychotherapist that the patient can develop greater awareness of their own inclinations, vocations, and relational traits, along with the challenges and the vulnerabilities that emerge when such traits interact with various forms of otherness (61). This in turn enables the patient to investigate the type of relational dynamic that they find beneficial or desirable, and to pursue relational experiences with awareness, assertiveness, and maturity.

## Author contributions

BB, CB, and PB: conceptualization, methodology, writing—draft, and writing—review and editing. All authors contributed to the article and approved the submitted version.

## Funding

This study was funded through the Fondazione Regionale Ricerca Biomedica under the Marie Skłodowska-Curie Actions Seal of Excellence Fellowship (ID 2681424) awarded to BB. This study was also supported by the Italian Ministry of Health (Ricerca Corrente 2022).

## Conflict of interest

The authors declare that the research was conducted in the absence of any commercial or financial relationships that could be construed as a potential conflict of interest.

## Publisher's note

All claims expressed in this article are solely those of the authors and do not necessarily represent those of their affiliated organizations, or those of the publisher, the editors and the reviewers. Any product that may be evaluated in this article, or claim that may be made by its manufacturer, is not guaranteed or endorsed by the publisher.
